# Vacuolar iron export alters the synergy between doxycycline and fluconazole by affecting cidal ROS levels in *Candida albicans*

**DOI:** 10.1128/mbio.00416-26

**Published:** 2026-04-20

**Authors:** Wouter Van Genechten, Michelle Holtappels, Martine De Jonge, Rudy Vergauwen, Gauthier Cornips, Patrick Van Dijck

**Affiliations:** 1 Laboratory for Molecular Cell Biology, Department Biology, KU Leuven 26657, Leuven, Belgium; 2 One Health institute, KU Leuven 26657, Leuven, Belgium; Universidade de Sao Paulo Campus de Ribeirao Preto, Ribeirao Preto, Sao Paulo, Brazil

**Keywords:** iron transport, iron regulation, antifungal therapy, mitochondrial metabolism, fluconazole, doxycycline, *Candida albicans*, vacuoles

## Abstract

Fungal infections are combatted using three main classes of antifungals, of which the azoles, considered to be fungistatic, are the most widely used. Slow growth of *Candida albicans* at supra-minimal inhibitory concentrations (MIC) of fluconazole (FLC), termed tolerance, is routinely observed. A combination therapy resulting in the eradication of this fungistatic character would be a valid therapeutic strategy, and indeed, the synergistic combination of the antibiotic doxycycline and FLC has such an effect. We hypothesized that iron-requiring mitochondrial functions may be the targets of the synergistic combination. The proteome enriched for mitochondria obtained from FLC + Fe-treated cells hinted that iron alleviated the FLC stress and that intracellular iron homeostasis, more specifically the vacuolar iron exporter Smf3, might be a key factor during FLC treatment, as its expression was induced. Moreover, a ROS assay revealed that a *smf3Δ/Δ* strain treated with FLC accumulated ROS to a similar extent as that displayed by the WT undergoing a FLC+DOX combination treatment. Thus, deletion of *SMF3* mimics the addition of doxycycline in wild-type cells. The ROS accumulation can be attenuated through overexpression of the mitochondrial superoxide dismutase *SOD2,* and this restored the synergy between DOX and FLC in the *smf3Δ/Δ* background. ROS accumulation, in part through altered iron availability from the vacuolar storage pool, is thus the molecular mechanism underlying the synergy between doxycycline and FLC. Furthermore, no effect on either cidality or tolerance was observed in the *smf3Δ/Δ* strain, highlighting that synergy is not necessarily an indication of cidal therapies.

**IMPORTANCE:**

Azoles are widely used against *Candida albicans*, yet many cells survive above the minimal inhibitory concentrations (MIC) by growing slowly, which can prolong infection and foster resistance. We show that intracellular iron homeostasis alters the fluconazole characteristics by affecting ROS accumulation in mitochondria, and that this is the molecular mechanism underlying the combination therapy of fluconazole and doxycycline. These results place iron release from the vacuole at the center of azole responses, suggesting novel ways to boost azole efficacy.

## INTRODUCTION

The fungal genus *Candida* comprises species that are the fourth most common cause of nosocomial systemic infections in the United States, with crude mortality rates of up to 40% (1). The most common pathogen within *Candida* spp. is *Candida albicans*, an opportunistic pathogen but also a member of the healthy microbiota (2). The high mortality is attributed to an increased population at risk, as well as increased antifungal drug resistance, mainly to the azole antifungal drug class. Even though echinocandins gradually replace fluconazole (FLC) as the first-choice treatment in the high-income industrialized countries, FLC still is the most widely used azole antifungal drug in many parts of the world (3, 4). Fluconazole targets the heme-containing Erg11, an essential enzyme involved in ergosterol biosynthesis. Rapid onset of resistance to FLC is caused through upregulation of drug efflux pumps (mainly by a set of constitutively active transcription factors), overexpression of *ERG11*, mutations within *ERG11* that affect the binding of azoles, and compensatory pathways for alternative sterol production, coupled with the induction of stress response pathways (5, 6). It is proposed that this rapid resistance development is caused by the fact that FLC is a fungistatic drug for *C. albicans* (7). In fact, most strains of *C. albicans* can grow slowly in the presence of supra-minimal inhibitory concentrations (MIC) of FLC, termed tolerance or fraction of growth (8, 9). The molecular basis for this phenotype is still unclear but seems to be linked with general stress response pathways and the pH-regulated Rim101 pathway (10, 11). Additionally, the amount of iron available to the pathogen is important for its tolerance against FLC. Adding doxycycline in combination with FLC completely prevents this tolerance and converts FLC from a fungistatic into a fungicidal drug (7). It was verified that the iron-chelating activity of doxycycline caused the synergistic effect, as the addition of iron could restore the fungistatic character (7). The important role of iron regarding FLC tolerance was also shown with other iron chelators and mutants in iron transporters (12, 13).

Iron acquisition in general is an important virulence factor for *C. albicans*, especially in certain iron-restricted niches, such as the bloodstream (14). This is partly due to a host defense mechanism, known as nutritional immunity (15). Therefore, *C. albicans* has developed sophisticated systems to scavenge iron within the host for survival. These mechanisms include a high-affinity reductive uptake system, a siderophore uptake system, and the hemoglobin-iron uptake system (14, 16–18). As high levels of iron become toxic for the cells, Fe^3+^ is stored in the vacuole, forming a complex with polyphosphate. Specific vacuolar iron importers, such as Ccc1, are involved in this process. To redistribute vacuolar iron, different reductases are involved in converting Fe^3+^ into Fe^2+^, which the vacuolar iron exporter Smf3 can then transport. Iron can then be transported into other organelles, including mitochondria, through Mrs4. This intracellular iron-homeostasis pathway, Mrs4-Ccc1-Smf3, was shown to be essential for mitochondrial function, virulence, and echinocandin resistance (19). However, the link between iron chelation and loss of tolerance or synergy with FLC was not investigated in the context of the intracellular iron pathway and mitochondria. In this study, we set out to identify key mitochondrial proteins during fluconazole treatment. Proteomics analysis was performed on extracts, which were enriched in mitochondrial proteins, that were isolated from cells grown in the absence or presence of FLC with or without the addition of iron. Fluconazole treatment led to a downregulation of several mitochondrial iron utilization processes, such as heme and iron-sulfur cluster biosynthesis (Hem1, Ssc1) and mitochondrial activity in general, as exemplified by downregulation of Aco1, a member of the TCA cycle. Although considered to be a vacuolar membrane protein, Smf3 was significantly upregulated in the presence of FLC in our proteomics approach, and deletion of the encoding gene resulted in a loss of synergy between fluconazole and either doxycycline or the iron chelators bathophenanthrolinedisulfonic acid (BPS) or bathophenantroline (Bphen). We established that deletion of *SMF3* results in a rapid ROS accumulation, which can be partially reversed through overexpression of the mitochondrial superoxide dismutase, encoded by *SOD2*. Concurrently, overexpression of *SOD2* in the *SMF3* deletion strain restored the synergistic character of doxycycline and fluconazole. When *C. albicans* is grown in hypoxic conditions, no DOX+FLC synergy or cidality is observed, further confirming the role of ROS in these phenomena. Interestingly, deletion of *SMF3* did not alter the tolerance profile (or the cidal response) to fluconazole alone or in combination treatment, highlighting that these synergistic combinations do not necessarily correlate with a loss of tolerance.

## RESULTS

### Abundance of mitochondrial and metal-binding associated proteins is significantly altered upon fluconazole treatment

**Fig 1:**
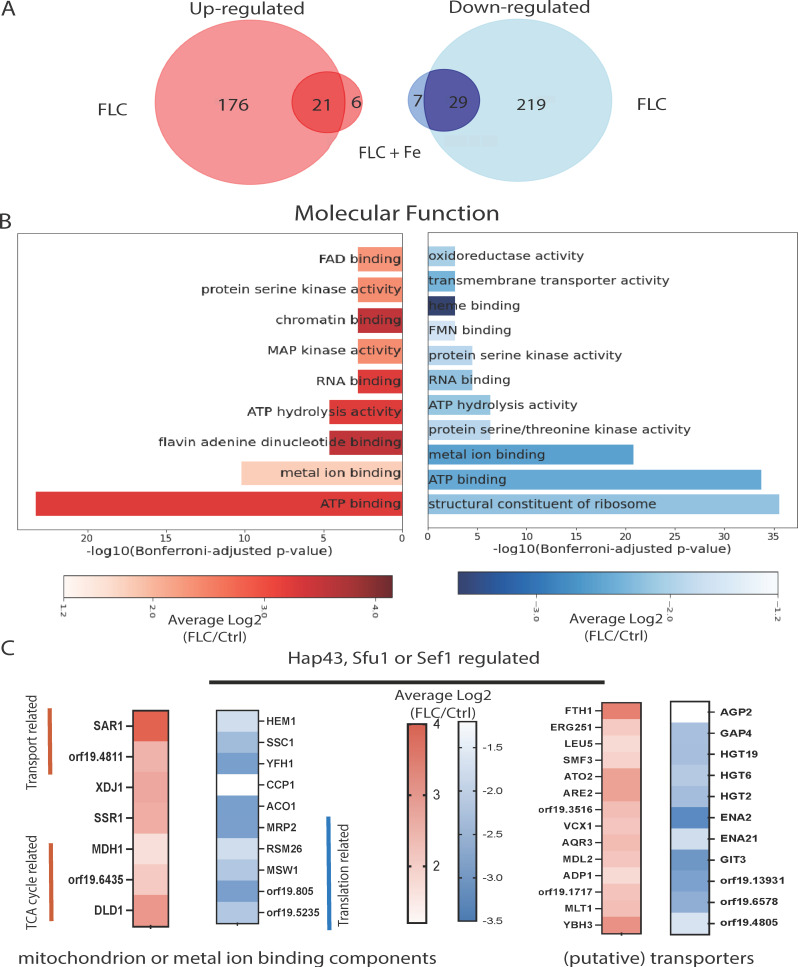
Abundance of mitochondrial and metal-binding associated proteins is significantly altered upon fluconazole treatment. (**A**) Venn diagram showing the unique and overlapping up- and down-regulated proteins during treatment with 8 µg FLC and with the conditional supplementation of 1 mM Fe_2_SO_4_. (**B**) GO ontology analysis of the molecular function of the up- and down-regulated proteins that are unique in response to the FLC stress. (**C**) Further filtering of the FLC-only responsive proteins based on whether they are related, either directly or indirectly, to the iron regulon. Some highlights are depicted here, which are selected based on the prominent molecular functions defined in panel B and putative transporters.

As fluconazole resistance, iron availability, and mitochondria are intricately linked (18), we assessed the proteome of an enriched mitochondrial fraction of cells undergoing fluconazole treatment with additional supplementation of iron. FLC treatment leads to a strong effect on the proteome, with 197 up- and 248 down-regulated proteins, respectively. Supplementation of iron during fluconazole stress results in a decrease in these proteome alterations, with only 27 and 36 significant up- and down-regulated proteins (Fig. 1A). The 176 and 219 proteins, of which the abundance is altered uniquely in the FLC condition, provide a means to investigate those processes that are heavily required during FLC stress but are obsolete when ample iron is present. A Gene Ontology (GO) assessment of molecular function from this subset reveals that ATP binding and metal ion binding are two main regulated processes (Fig. 1B). Interestingly, FLC treatment affects the expression of different transporters, as well as metal ion binding proteins, all of which are regulated by the iron regulon consisting of Hap43, Sfu1, or Sef1 (Table S1; Fig. 1C). Several of these proteins are still uncharacterized. The downregulation of three glucose transporters indicates that a process akin to the a-specific azole import by the *C. glabrata* hexose transporters may occur in *C. albicans* (20). Additional interesting proteins that are significantly less abundant during FLC treatment are Hem1, a heme biosynthesis enzyme, and Ssc1, a mitochondrial iron-sulfur cluster biosynthesis chaperone, illustrating that some iron utilization processes are at least partially limited. This does not indicate that the iron requirement of the mitochondria is also downregulated. Aconitase, encoded by *ACO1*, a regularly used marker for mitochondrial activity and part of the TCA cycle, also displays a lower abundance in FLC-treated cells. Additional factors reinforcing the alteration in iron homeostasis during FLC treatment are the downregulation of the putative (based on homology with *S. cerevisiae*) iron storage protein Yfh1 and the upregulation of the vacuolar iron exporters Smf3 and Fth1. Even though a large set of differentially abundant proteins is identified during this experiment, the focus of further research within this manuscript will be on the intracellular iron homeostasis pathway since upregulation of the vacuolar iron exporter Smf3 hints at a redistribution of cellular iron, which could then be utilized by the mitochondrial iron-requiring processes (e.g., Hem1, Ssc1, and Aco1).

### The vacuolar iron exporter Smf3 is required for the doxycycline-fluconazole synergy but has no effect on fluconazole sensitivity

**Fig 2:**
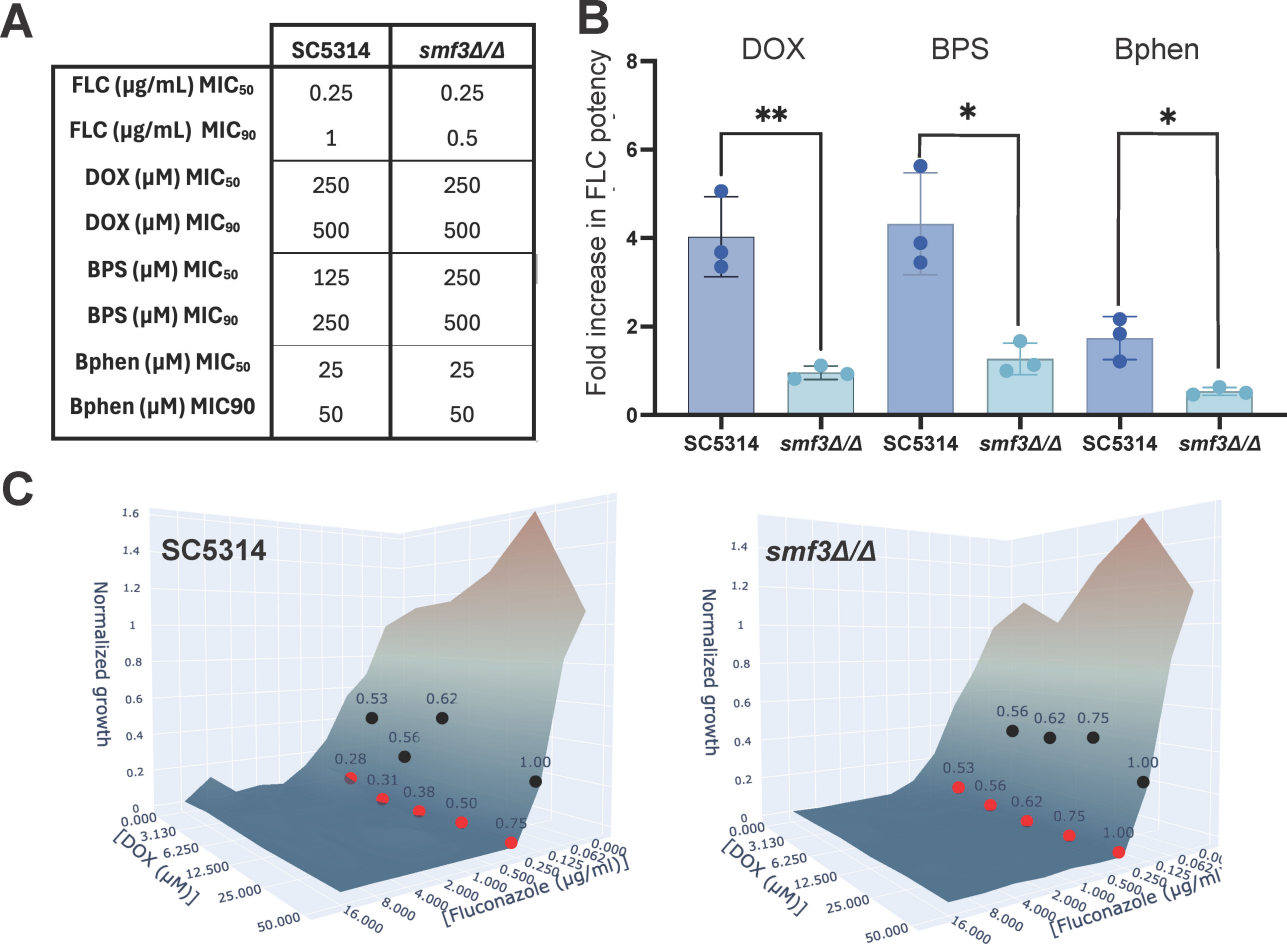
The intracellular iron homeostasis pathway alters the doxycycline-fluconazole synergy without affecting sensitivity. (**A**) MICs of FLC, DOX, and BPS at 50% and 90% inhibition. (**B**) MuSyC analysis from the checkerboard assays results in a potency increase metric. Data are presented as mean with SD. (**C**) Growth profiles of checkerboard assays where values are normalized to the wells where no drugs are administered. On top of these growth profiles, the round markers indicate iso-inhibition zones with their fractional inhibitory concentration (FIC). Black, yellow, and red dots indicate 50%, 80%, and 90% inhibition, respectively. If two metrics, for example, 80% and 90% overlap, the more stringent marker is depicted. Statistical significance is indicated using asterisks as follows: *P* < 0.05 (*), *P* < 0.01 (**), *P* < 0.001 (***), *P* < 0.0001 (****).

Smf3, the vacuolar iron exporter that is upregulated in the presence of FLC, is part of the intracellular iron homeostasis pathway consisting of Mrs4, Ccc1, and Smf3. Therefore, the role of this pathway in the synergy between iron chelation and FLC was further explored using broth dilution (BDA) and checkerboard assays. The MIC listed in Fig. 2A highlights that deletion of *SMF3* renders the strain not significantly more sensitive or resistant to the antifungal fluconazole, the antibiotic doxycycline, the iron chelator BPS, or the cell-permeable iron chelator Bphen. Additionally, deletion of other components of the intracellular iron homeostasis pathway also does not alter MICs (Fig. S1A).

However, as mentioned before, the doxycycline-fluconazole synergy is based on the iron chelation effect of doxycycline. Therefore, we tested whether disrupting part of vacuolar iron export affects this synergy. We utilized a synergy model termed MuSyC to quantitatively assess synergy (21). Within this model, we selected the potency parameter, which provides a quantitative assessment of the fold increase in potency the adjunct has on FLC sensitivity (Fig. 2B). Direct comparison of this potency index with the wild type confirms the absence of a potent interaction between fluconazole and DOX, BPS, or Bphen in the *smf3Δ/Δ* compared to the wild type (*P* = 0.0059, *P* = 0.0069, *P* = 0.0135, respectively). Vacuolar iron export is thus an (in)directly required process during the synergistic interaction between iron chelation + FLC treatment. In order to confirm the potency and assess the absence of synergy of the *SMF3* deletion strain, we also utilized the classical fractional inhibitory concentration (FIC) index as a metric. The checkerboard assays displayed in Fig. 2C illustrate the response profile of the strains with their FIC index indicated at the 50%, 80%, and 90% isoinhibition line (i.e., the line connecting all locations in the two-dimensional checkerboard assay where an inhibition of, for example, 50% is obtained). It is apparent that for the SC5314 wild-type strain, the multiple FIC90 indices listed at the 90% isoinhibition line are lower than 0.5, thus reporting a synergistic relation between FLC and DOX. Interestingly, in the *smf3Δ/Δ* strain*,* none of the FIC90 indexes are lower than 0.5, indicating an absence of the FLC+DOX synergy. Deletion of other components of the intracellular iron homeostasis pathway did not affect synergy (Fig. S1, panels B and C). These results are mirrored in an FLC+BPS and FLC+Bphen checkerboard assay, of which the results are displayed in Fig. S2, panels A and B, respectively.

### FLC and DOX results in ROS accumulation, and the *SMF3* deletion strain hyperaccumulates mitochondrial ROS

**Fig 3:**
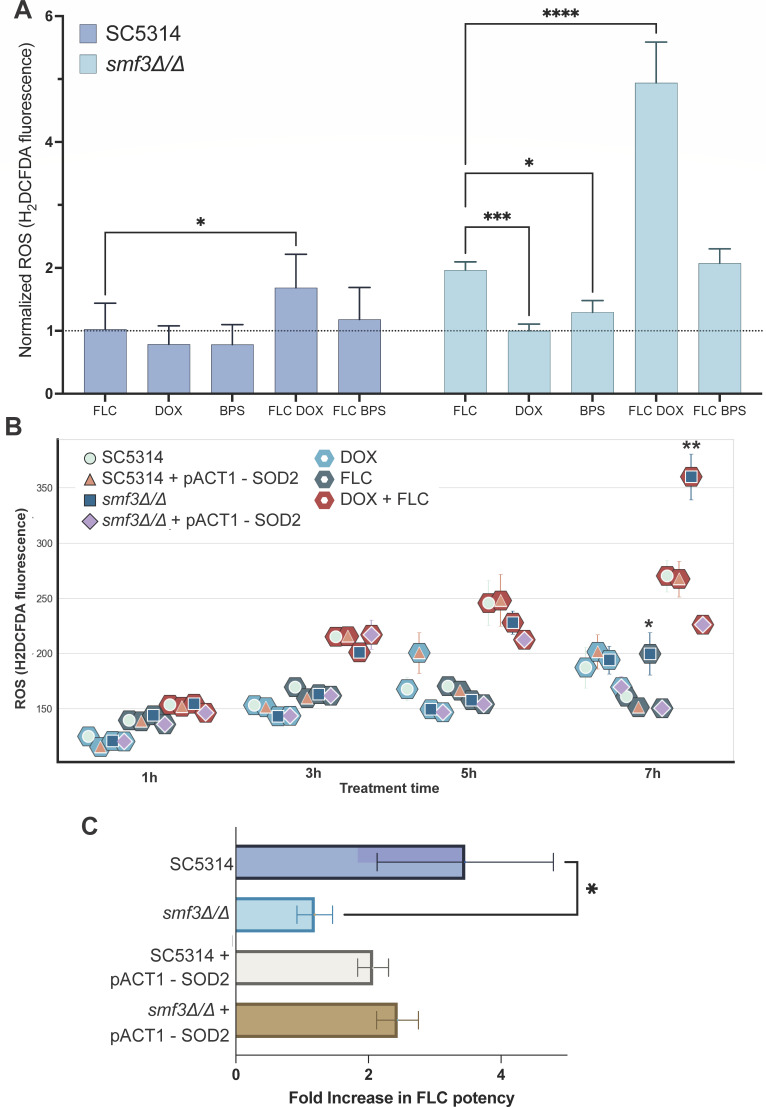
FLC and DOX results in ROS accumulation, and the *SMF3* deletion strain hyperaccumulates mitochondrial ROS. (**A**) Relative ROS quantity measured through the addition of the ROS-reactive dye H_2_DCFDA to the WT and the *smf3Δ/Δ* after 24 h of FLC, DOX, BPS, FLC+DOX, or FLC+BPS treatment, normalized to the no-treatment control condition. Data are presented as mean with SD. (**B**) Longitudinal ROS assay. Data are presented as the mean with SD of *n* = 3 per sample and condition. (**C**) MuSyC analysis from the checkerboard assays results in a potency increase metric. Data are presented as mean with SD. Statistical significance is indicated using asterisks as follows: *P* < 0.05 (*), *P* < 0.01 (**), *P* < 0.001 (***), *P* < 0.0001 (****).

To understand the underlying molecular mechanism behind the iron chelation + FLC treatment synergy, we assessed whether the minor ROS production during fluconazole treatment is aggravated in the *SMF3* deletion strain, the only iron homeostasis mutant where synergy was disrupted. Fluconazole and azoles in general are considered fungistatic. An exception to the fungistatic character of azoles is itraconazole, which induces mitochondrial dysfunction and ROS accumulation, resulting in a cidal therapy against *C. albicans* (22). This ROS accumulation, without a direct link to cidality, is also observed for miconazole and to a smaller extent for fluconazole (23, 24). To assess whether ROS accumulation contributes to the synergistic character of the combination therapies, we assessed whether single and combination treatments induce ROS production as measured through H_2_DCFDA oxidation. Figure 3 panel A depicts the results from these ROS measurements, and it can be appreciated that after 24 h, there is an approximate 1.5-fold increase in ROS in the wild type subjected to the FLC+DOX combination therapy compared to FLC alone (*P* = 0.0239). A strong effect was observed in the *SMF3* deletion strain, where the FLC-alone condition resulted in a twofold increase in ROS compared to the no-treatment condition. Furthermore, the combination therapy of FLC+DOX in the *smf3* mutant resulted in a vast accumulation of ROS compared to the already elevated ROS levels in the FLC-alone condition (*P* < 0.0001). Interestingly, doxycycline or BPS alone did not induce any ROS level in the WT or *smf3∆/∆* strain. To determine whether the *smf3Δ/Δ* strain accumulates this ROS within the mitochondria, we overexpressed a mitochondrial superoxide dismutase (Sod2) and subjected the strains to a kinetic ROS assay (Fig. 3B). After 1 h, the ROS levels in the three treatment conditions (doxycycline, fluconazole, or the combination of these) are still similar, with a minor shift toward higher ROS in the combination treatment. However, the strains (SC5314 with or without overexpressed *SOD2*, and *smf3Δ/Δ* with or without overexpressed *SOD2*) are still closely grouped together. After 3 h, no real ROS accumulation is observed for single treatments, and this is also the case after 5 h. However, the FLC+DOX combination therapy does result in ROS accumulation, and from 5 h onward, this difference is significant. At 7 h, within the FLC+DOX condition, an effect of the *SOD2* overexpression can be observed. Similar to the ROS profile in Fig. 3A, the *SMF3* deletion strain accumulates ROS, and these levels are significantly reduced when overexpressing the mitochondrial superoxidase *SOD2* in that background (*P* = 0.017). This significant increase in ROS of the *SMF3* deletion strain is also present in the FLC condition (*P* = 0.007). All statistical comparisons are available in Table S2, and untreated controls are included in Fig. S3, panel A. To assess whether *ACT1* promoter activity is not significantly different between strains under the drug conditions, we performed qPCR, of which the results are shown in Fig. S3, panels B and C.

As described earlier, Smf3 is significantly increased in expression in the proteomics data under FLC treatment, and deleting *SMF3* results in the absence of the synergy of DOX or BPS with FLC. We therefore investigated whether overexpression of *SOD2* alters the FLC+DOX synergy. Similar to the results shown in Fig. 2B, the results in Fig. 3C depict that the wild-type strain SC5314 is subject to the synergistic combination of DOX and FLC (fold increase in potency of 3.46), whilst this potent synergy is absent in the *smf3* mutant (*P* = 0.0109, fold increase in potency of 1.19). Overexpression of SOD2 in the wild-type strain does not significantly alter the potency index, but overexpression of SOD2 in the *smf3Δ/Δ* strain does result in a synergy restoration with an increase in the potency of FLC to 2.44. MuSyC potency index with 95% CI for these strains is listed in Table S3. The FIC_90_ results confirm the synergy results and are depicted in Fig. S4.

The underlying molecular mechanism for the FLC+DOX synergy is thus the inability to export vacuolar iron, resulting in a mitochondrial ROS accumulation which can be partially counteracted through overexpression of a superoxide dismutase. While iron chelation does result in a synergistic therapy, it does not induce an ROS accumulation; an additional role of DOX must be present other than simply chelating iron.

### Smf3-related ROS accumulation does not result in an enhanced cidal fluconazole therapy

**Fig 4:**
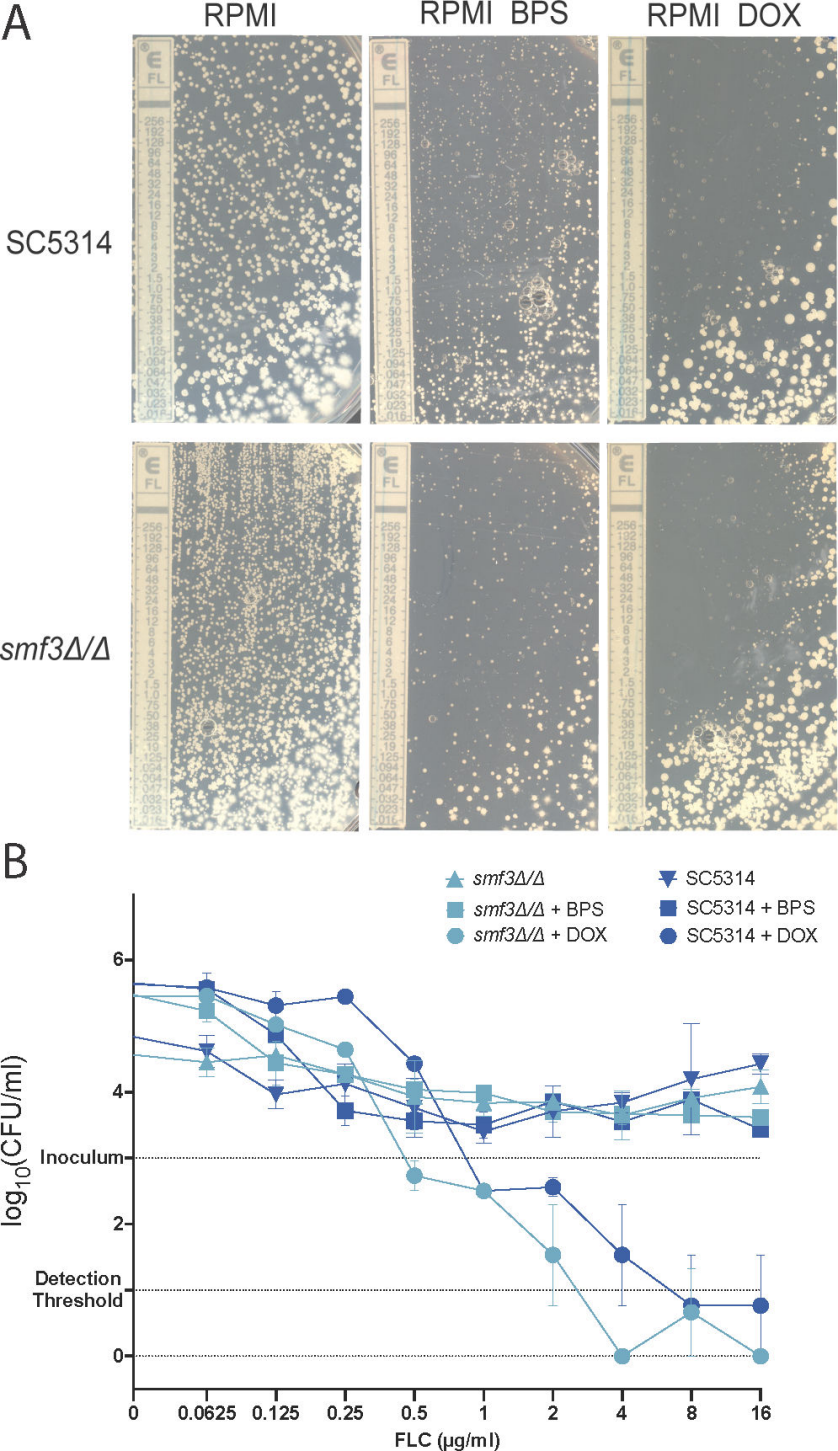
Smf3-related ROS accumulation does not result in a cidal fluconazole therapy. (**A**) E-test (BioMérieux) strips containing a gradient of FLC were placed on RPMI 1640 agar plates containing a lawn of *C. albicans* cells. RPMI 1640 was also supplemented with either 50 µM BPS or DOX. Tolerance of the strains is evaluated by assessing the appearance of slow growth within the inhibition halo. (**B**) Survival curves of *C. albicans* strains after 48 h of treatment with FLC in combination with DOX or BPS as measured through viable CFU counting. The two different strains are represented as distinct colors, while the treatment scheme—DOX, BPS, or no additional treatment—is shown as dots, squares, or triangles, respectively. Data is represented as the mean and SD of three independent biological transformants.

As mentioned earlier, the ROS assays were performed to measure whether a combination therapy resulting in ROS would result in a cidal and/or non-tolerant therapy, similar to itraconazole and miconazole therapy. A common readout for tolerance is assessing growth within the inhibition zone of an E-test strip, as depicted in Fig. 4, panel A. When *C. albicans* is subjected to an E-test strip containing a gradient of FLC, we can clearly see that the halo contains growth in the RPMI condition. When we supplemented the RPMI medium with 50 µM BPS, both the wild type and the *smf3Δ/Δ* still displayed, albeit less dense and apparent, some growth. As expected, the combination of an FLC E-test with a doxycycline-containing medium removed tolerance for the wild type and the *smf3Δ/Δ* strains. The absence of tolerance only indicates that cells are unable to grow to a large enough density for visual assessment and does not necessarily suggest that cells were killed through a cidal mechanism. Therefore, in a liquid BDA assay (Fig. 4B), we assessed the effect of BPS and DOX on growth and survival. Without DOX or BPS, the strains grow and survive at supra-MIC concentrations of FLC. The combination of an FLC dilution series with 50 µM of BPS resulted in tolerant and growing wild type and *smf3Δ/Δ* cells, while 50 µM of DOX resulted in a cidal therapy. Both the wild type and the *smf3Δ/Δ* strains display very similar survival curves, suggesting that the extensive ROS accumulation in the *smf3Δ/Δ* strain does not result in a stronger cidal therapy of either FLC or FLC with an iron chelator compared to the wild type. Intracellular iron chelation by Bphen does not result in a cidal therapy (Fig. S5), supporting that the iron chelation properties of doxycycline are not the only molecular mechanism underlying its cidality.

### DOX-induced cidality and synergy are absent in anaerobic conditions

**Fig 5:**
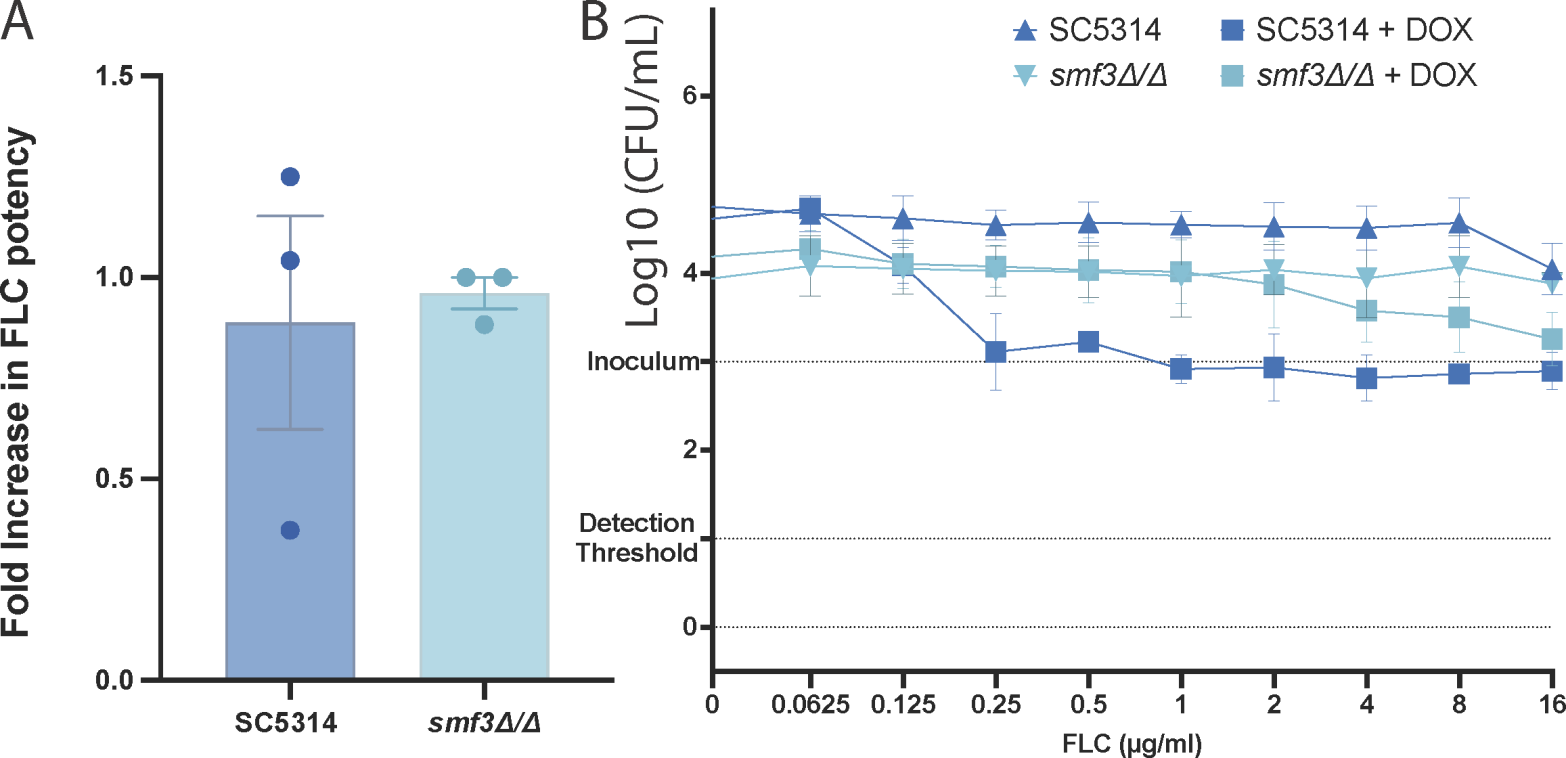
DOX-induced cidality and synergy are absent in anaerobic conditions. (**A**) Potency increase metrics for wild type and *SMF3* deletion strains under anaerobic conditions. MuSyC analysis indicates these values are not significantly different from one as determined by a one-sample *t*-test. (**B**) Survival curves of wild type and *SMF3* deletion strain after 72 h of treatment with FLC in combination with DOX in anaerobic conditions as measured through viable CFU counting. The two different strains are represented as distinct colors, while additional treatment (DOX) or no additional treatment is shown as squares or triangles, respectively. All data are presented as mean with SD for three independent biological repeats.

We next set out to assess whether, under anaerobic conditions, thus limiting the theoretical capacity to produce ROS, the synergy and cidality of the DOX+FLC combination therapy are still present. From Fig. 5, panel A, it is apparent that the DOX+FLC combination therapy is no longer synergistic under anaerobic conditions against either the wild type or the *SMF3* deletion strain. The fold increase in potency is non-significantly different from the theoretical value of 1 as assessed through a one-sample *t*-test and is not significantly different between the two strains. These results are mirrored in the FIC measurements, which are depicted in Fig. S6. Additionally, even though overall growth was limited due to the hypoxia (comparing Log_10_ CFU/mL values with those from Fig. 4, panel B), no cidal effect is observed. Even the combination treatment of DOX+FLC did not result in a vast drop in viable cells below the original inoculum. The (near) absence of oxygen thus restored viability of the cells at supra-MIC conditions of FLC in the presence of DOX.

## DISCUSSION

It is generally recognized that iron homeostasis of *Candida albicans* is a key factor during host infection, and that this species has ample strategies to acquire this micronutrient. A proteomics experiment to identify iron-related factors required during fluconazole treatment of *C. albicans* highlighted that supplementation of iron (in conjunction with FLC) resulted in approximately sevenfold lower number of proteins up- or down-regulated compared to the FLC treatment alone. This indicates that adequate iron availability partially alleviates the FLC-induced stress. That iron availability is tightly linked to FLC tolerance was already reported by our laboratory, as we previously showed that a combination treatment of doxycycline, which has iron chelating properties, renders fluconazole fungicidal (7). It was also known that deleting components of the iron reductive pathway resulted in increased susceptibility to azoles (13).

Even though ferrous iron is essential for numerous cellular processes and increases resistance to azoles, excessive quantities are toxic due to its tendency to oxidize into ferric iron, producing harmful amounts of ROS as a side product. To prevent toxic levels of iron within the cytosol, most of it is safely stored inside the vacuole and distributed across organelles by the three intracellular iron transporters Mrs4, Ccc1, and Smf3 (19). It was therefore interesting that in our proteomics experiment, we identified many proteins involved in iron transport, utilization, and storage which had altered abundances. Among these was Smf3, a vacuolar iron exporter that was significantly upregulated during FLC treatment, but not when additional iron was supplemented. Even though our proteomics of mitochondria-enriched fractions did result in the identification of mitochondria as one of the main cellular components through GO analysis, it is clear that many non-mitochondrial transporters were identified. These were most likely co-extracted due to the close contact between organelles through inter-organelle contact sites, such as the vacuole and mitochondria patch termed vCLAMP (25). These contact sites are considered to enhance protein, lipid, and iron transport, thereby explaining the extraction of Smf3 (26–28).

Since Smf3 is part of the intracellular iron homeostasis, we set out to fully characterize this pathway in order to further increase our understanding of fluconazole susceptibility and tolerance. Whereas deleting any of these transporters did not result in a significant alteration of susceptibility toward fluconazole, doxycycline, BPS, or Bphen, we identified that deletion of *SMF3* results in an absence of the synergy between fluconazole and the iron chelators. This suggests that individual susceptibility profiles (MICs) cannot be directly linked to enhanced synergy. As highlighted in the study by Fiori and Van Dijck, the synergistic combination of fluconazole and doxycycline altered the fungistatic aspect of azoles, resulting in a cidal therapy (7). We reconfirmed that doxycycline indeed removes tolerance to azoles, but this is contrasted with the observation that BPS or Bphen does not alter fluconazole tolerance of the *C. albicans* wild type. This phenomenon, together with the synergy assay results, suggests that both intracellular and extracellular iron chelation does increase fluconazole potency but is not solely responsible for cidality. This clearly suggests that another aspect of doxycycline is also required for this effect.

Not all azoles are considered fungistatic, and itraconazole is such an exception. Itraconazole treatment of *C. albicans* triggers mitochondrial dysfunction, ROS accumulation, and subsequent apoptosis. Interestingly, this ROS accumulation is proposed to happen through the disruption of iron homeostasis (22). Other azoles were also reported to produce ROS, such as miconazole, and the ROS produced during this therapy resulted in apoptosis and is therefore considered a fungicidal azole (23, 29). Fluconazole was also shown to induce some ROS in *C. albicans* and *Cryptococcus neoformans*, although to a lower extent (23, 30). It is therefore not fully established that ROS is the main determinant of a fungistatic or fungicidal outcome, and its production is most likely azole concentration-dependent. ROS measurements during FLC treatment or from the combination therapies tested throughout this study support the fact that FLC by itself does not result in large accumulations of ROS. However, the combination of FLC and DOX did result in such an increase. Interestingly, the *SMF3* deletion strain did accumulate ROS in the FLC-alone condition, and these levels further increased when DOX was also administered. However, the *SMF3* deletion strain does not display an enhanced cidal profile to any combination therapy. This could indicate that the extent of ROS in the wild type is already sufficient to result in a cidal therapy, and that the extensive levels in the *SMF3* deletion are superfluous. The only susceptibility characteristic in which the *smf3Δ/Δ* is affected is the synergism. Combined with the observation that the FLC-alone condition resulted in enhanced ROS, it can be hypothesized that deletion of *SMF3* mimics the effect of doxycycline during a combination treatment with FLC. The *SMF3* deletion strain thus effectively loses the dose-dependent sensitivity to DOX required for FIC or MuSyC synergy calculations, thereby masking the synergy of this combination therapy. This ROS-driven susceptibility is likely threshold-dependent, appearing primarily at high FLC drug pressures as evidenced by the observed minor shift of the mutant at MIC_90_ rather than at MIC_50_.

To further verify the link between ROS, synergy, and cidality, we overexpressed a mitochondrial superoxide dismutase and assessed ROS accumulation longitudinally. It is immediately apparent that the FLC+DOX combination results in ROS accumulation, which is the strongest in the absence of Smf3, but this accumulation is reduced when *SOD2* is overexpressed. Additionally, the combination of *SMF3* deletion with *SOD2* overexpression restored synergy. The capacity of doxycycline to induce mitochondrial ROS is thus one of the molecular mechanisms underlying the synergy of FLC+DOX, the other being the general iron chelation, since the BPS treatment does not result in ROS. In the relatively new fungal pathogen *Candida auris,* a similar increase in ROS levels is observed during treatment with the synergistic combination of itraconazole and the antiemetic aprepitant (31). This treatment also interfered with metal ion transport in *C. auris*, thereby suggesting that metal ion homeostasis and subsequent ROS accumulation is a molecular mechanism present throughout the *Candida* clade. In *C. albicans,* iron is not only important during fluconazole treatment, but it also binds caspofungin and diminishes antifungal activity (32). Additionally, assessing synergy and cidality under anaerobic conditions highlights that in the absence of the capacity to produce ROS, both DOX+FLC synergy and cidality are abolished.

Together, our data show that elevated ROS, driven in part by restricted access to vacuolar iron, underlies the cidality between doxycycline and fluconazole. Iron chelation alone (intra- or extracellular) is synergistic with FLC but does not alter ROS levels and therefore does not increase fungicidal activity. The lack of effects on cidality or tolerance in the *smf3Δ/Δ* strain, whilst synergy is affected, demonstrates that pharmacologic synergy can emerge through non-cidal mechanisms, a nuance that should guide interpretation of antifungal combination therapies.

## MATERIALS AND METHODS

### Strain construction and growth conditions

All strains utilized in this study (as listed in Table S4) were routinely cultured on Yeast Extract Peptone medium containing 2% glucose (YPD). Unless stated otherwise in the material and methods section, overnight cell cultures were grown in 3 mL of YPD at 30°C. Since the experiments related to the mitochondria were designed to mimic the conditions of the mitochondrial isolation, these were performed with galactose as a carbon source. Anaerobic growth was achieved through placement of the 96-well plates in sealed chambers with Anaerocult bags, and the effectiveness of the anaerobic environment was assessed through Anaerotest strips with an effective anaerobic environment of <0.1% O_2_.

CRISPR-Cas9 was utilized to construct the deletions in the intracellular iron homeostasis pathway based on the method by Nguyen et al. (33), which utilizes gRNA-specific primers that are listed in Table S5. After the transformation of all CRISPR-Cas9 materials in the SC5314 parent strain based on the heat shock transformation as described by Gietz et al. (34), strains were selected on YPD containing Nourseothricin. Correct removal of the ORF was checked through two PCR reactions: one containing both primers up- or downstream of the ORF, and one reaction with a primer within the ORF. All primers are listed in Table S5. After selecting three correct deletion strains, individual colonies were grown on YP maltose (2%) to induce the flippase and subsequent removal of the nourseothricin marker and the *CAS9*.

To create the overexpression of the *SOD2* ORF, we cloned the ORF into the CIp10 vector (35) by restricting the vector with Pst1 and Nhe1 and creating an insert with overhanging tails through a PCR product with primers targeting the *SOD2* ORF in genomic DNA. Gibson assembly of these two linear fragments resulted in an *SOD2* containing CIp10 plasmid, which, through restriction with Stu1, is suitable for genomic integration at the *RPS10* locus of *C. albicans*. Integration of this plasmid was checked through a colony PCR with a forward primer in the *ACT1* promoter and a reverse primer in the *SOD2* ORF.

### Mitochondrial isolation

After a preculture of 7 h, the cells were diluted to an OD600 of 0.05 in 250 mL SC medium containing 2% galactose, and optionally with 8 µg/mL fluconazole and 1 mM Fe_2_(SO_4_)_3_. These cultures were incubated for 16 h at 30°C. Afterward, cells were collected by centrifugation at 4,500 rpm for 5 min and washed using sterile MilliQ. The cell pellet was resuspended in Tris-SO_4_ (10 g wet weight corresponds to 20 mL buffer) before a 20-min incubation at 30°C. After centrifugation (5 min, 4,500 rpm), cells were collected and washed in 40 mL sorbitol buffer before the final resuspension in sorbitol buffer (2× wet weight), and lyticase (0.25 mg/g wet weight) was added. This suspension was gently shaken (1 h, 70 rpm, 30°C) and centrifuged (5 min, 2,500 rpm) to harvest the spheroplasts.

The pellet was resuspended in SEM buffer (10 g wet weight corresponds to 10 mL buffer) and transferred into a tight dounce and was homogenized 15 times. This suspension was transferred into an SS34 ultracentrifuge tube and centrifuged (5 min, 5,000 rpm, 4°C). The supernatant was transferred to a new SS34 centrifuge tube, whilst the pellet was resuspended in SEM and transferred into a tight dounce for a second round of homogenization. This suspension was transferred and centrifuged as before (5 min, 5,000 rpm, 4°C), and the resulting supernatant was added to the previous supernatant in the SS34 centrifuge tube and centrifuged for 10 min at 10,000 rpm. The obtained cell pellet contained the crude mitochondrial fraction, and the supernatant was discarded. This cell pellet was resuspended in 10 mL SEH buffer and centrifuged for 10 min at 10,000 rpm. Finally, the pellet is resuspended in 1–2 mL of SEM buffer, depending on the size of the pellet. This is the crude mitochondrial fraction.

To further purify the crude mitochondrial fraction, we applied this extract onto a Histodenz gradient, ranging from 25% to 5%, which was prepared in Beckman 14 × 89 mm Ultra-Clear Centrifuge tubes. Gradients were made by first transferring 2 mL of the 25% Histodenz into an ultracentrifuge tube, subsequently overlaying this with 2 mL of the 20% Histodenz solution, and so forth until finally applying the 5% Histodenz solution. The gradient was kept at RT for 3–4 h in order to allow the gradient to diffuse. One hour before use, the gradient was chilled at 4°C. The crude mitochondrial fraction was applied onto the top of the gradient and centrifuged in a swinging bucket rotor (60 min, 100,000 × *g*, 4°C). Discontinuous density gradient centrifugation separated the different components based on their density. After centrifugation was completed, the bands can be removed sequentially, including the band with the purified mitochondria located at the 20%/15% interface. The mitochondrial fraction was transferred to a new, precooled tube using a Pasteur pipette. The Histodenz was removed by diluting the mitochondrial suspension five times in SEH buffer and subsequently centrifuging (10 min, 1,000 rpm, 4°C). After this final wash step, the pellet which contained purified mitochondria was dissolved in a sample buffer suited for proteome analysis (8 M urea, 20 mM Tris-HCl, pH 8). Finally, the mitochondria were subjected to sonication using a tip sonicator (8 × 10 s, with 10 s intervals between the pulses, 4°C) at 80% amplitude to break the mitochondria to obtain a homogeneous fraction for protein determination prior to the LC-MS/MS.

### Proteomics analysis

#### Sample preparation

Mitochondrial proteins were isolated, and the proteins were quantified. For each condition, three biological repeats were sent for LC-MS/MS analysis by the VIB proteomics core. Proteins in each sample were reduced by adding 4.5 mM DTT and incubated for 30 min at 55°C. Alkylation of the proteins was achieved by the addition of 10 mM iodoacetamide for 15 min at room temperature in the dark. Samples were diluted with 20 mM Tris-HCl, pH 8.0, to obtain a urea concentration of 4 M, and the proteins were digested with 0.5 µg lysyl endopeptidase (1/200, wt/wt) for 4 h at room temperature. All samples were further diluted with 20 mM Tris-HCl, pH 8.0, to a final urea concentration of 2 M, and proteins were digested with 1 µg trypsin (1/100, wt/wt) overnight at 37°C. Peptides were then purified with OMIX C18 pipette tips (Agilent) and vacuum dried.

#### LC-MS/MS and data analysis

The fractionated peptides were completely dried, dissolved in loading solvent A (0.1% FA in water/ACN [98:2, vol/vol]), and 3 µg-containing portions were injected for LC-MS/MS analysis. Samples were analyzed on a 40 cm reverse-phase column packed in the needle (produced in-house, 75 μm I.D. × 400 mm, 1.9 μm beads C18 Reprosil-HD, Dr. Maisch), using a non-linear 150 min gradient of 2%–56% solvent B (0.1% FA in water/ACN, 20:80 [vol/vol]) at a flow rate of 250 nL/min. This was followed by a 10 min wash reaching 99% of solvent B and re-equilibration with solvent A (0.1% FA in water). Column temperature was kept constant at 50°C (CoControl 3.3.05, Sonation). The Q Exactive HF mass spectrometer with a Nanospray Flex Ion source (Thermo Fisher Scientific) was operated in data-dependent, positive ionization mode, automatically switching between MS and MS/MS acquisition for the 16 most abundant peaks in a given MS spectrum. The source voltage was set to 2.5 kV and the capillary temperature was 250°C. MS1 scans (m/z 375–1,500, AGC target 3 × 106 ions, maximum ion injection time of 60 ms) acquired at a resolution of 60,000 (at 200 m/z) were followed by up to 16 tandem MS scans (resolution 15,000 at 200 m/z) of the most intense ions fulfilling predefined selection criteria (AGC target 1 × 105 ions, maximum ion injection time of 80 ms, isolation window of 1.5 m/z, fixed first mass of 145 m/z, spectrum data type: centroid, under fill ratio 1%, intensity threshold 1.3 × 104, exclusion of unassigned and singly charged precursors, peptide match preferred, exclude isotopes on dynamic exclusion time of 12 s). The HCD collision energy was set to 28% Normalized Collision Energy, and the polydimethylcyclosiloxane background ion at 445.12003 Da was used for internal calibration (lock mass).

Data analysis was performed with MaxQuant (version 1.5.6.5) using the Andromeda search engine with default search settings, including a false discovery rate set at 1% on both the peptide and protein level. Spectra were searched against the *Candida albicans* strain SC5314 proteins in the UniProt database (database release version of November 2016 containing 7,004 yeast protein sequences) (www.uniprot.org). The mass tolerance for precursor and fragment ions was set to 4.5 and 20 ppm, respectively, during the main search. Enzyme specificity was set as C-terminal to arginine and lysine (trypsin), also allowing cleavage at arginine/lysine-proline bonds with a maximum of two missed cleavages. Carbamidomethylation of cysteine was set as a fixed modification, and variable modifications were set to oxidation of methionine (to sulfoxides) and acetylation of protein N-termini.

Proteins were quantified by the MaxLFQ algorithm integrated into the MaxQuant software. Only proteins with at least one unique or razor peptide were retained, leading to the identification of 2,990 *Candida albicans* proteins. A minimum ratio count of two unique or razor peptides was required for the quantification of 2,186 proteins. Further data analysis was performed with the Perseus software (version 1.5.5.3) after loading the protein groups file from MaxQuant. Proteins only identified by site and reverse database hits were removed, as well as potential contaminants. The replicate samples were grouped. Proteins with less than three valid values in at least one group were removed, and missing values were imputed from a normal distribution around the detection limit, resulting in the protein list available in Data set S1.

### Broth dilution and drug cidality assay

To quantitatively determine the MIC of fluconazole, BPS, and doxycycline, a BDA was performed using a protocol based on both CLSI (36) and EUCAST (37) standard methods. UV-sterilized 96-well round-bottom microtiter plates were first prepared by filling them with 100 µL 2× RPMI 1640. A working stock solution of fluconazole (32 µg/mL), BPS (100 µM), and doxycycline (100 µM) was prepared in MilliQ before distribution to the next-to-last column in the 96-well plate. A twofold serial dilution is then pipetted, making sure that the second column of the plate does not receive any drug suspension. Finally, 100 µL of cell suspensions were loaded into these broth dilution plates. Growth was determined after 48 h of incubation at 37°C by measuring the OD at 600 nm after resuspending the cultures. MIC50 and MIC90 were determined based on where the OD values would drop below the 50% and 90% threshold compared to the background corrected OD of the no-treatment controls. For the checkerboard assay, plates were filled with 2× RPMI 1640, similar to the BDA assay. And stock solutions of all drugs were prepared and distributed across the 96-well plate according to the scheme reported by Bellio et al. (38). With the only adaptation that the wells on the periphery of the plate were not utilized, since we frequently observe evaporation and thus a skewed OD reading of these wells. Cells were inoculated in a fashion identical to the BDA. ∑FIC values were determined using the following formula, based on the CLSI guidelines and according to the Instructions to Authors of Antimicrobial Agents and Chemotherapy: ∑FIC = (MIC_chelator in combination_/MIC_chelator_) + (MIC_FLC in combination_/MIC_FLC_). Based on these guidelines, a ∑FIC of ≤0.5 implies synergy, a ∑FIC of >4 implies antagonism, and the values in between denote indifference. Both the ∑FIC for an MIC50 and an ∑FIC for an MIC90 were determined, based on MIC50 and MIC90 data.

To evaluate drug tolerance and cidality, we generated dose-response curves based on CFU counts for the control and/or mutant strain as described by CLSI and as established above. After 48 h of growth at 37°C, the contents of the wells of the checkerboard assays were resuspended, and each culture was diluted, plated, and incubated at 37°C before counting viable colonies. Anaerobic experiments were incubated for 72 h to have sufficient growth for optical density assessment. All experiments were conducted with at least three independent biological transformants.

### E-test assays

For the E-test assay, overnight cultures were adjusted to an OD600 of 0.2 in water and spread on RPMI 1640 agar medium. After placing the strip onto the cells, the plates were incubated for 72 h at 30°C. The MIC-FLC was determined by identifying the concentration of fluconazole on the strip where the latter intersects the halo of growth inhibition/retardation. The plates were scanned after 72 h of incubation at 37°C.

### ROS assay

The OD of overnight cultures grown in YPD was set at 0.3 and subsequently incubated in 3 mL SC 2% Gal at 30°C with shaking at 250 rpm until an OD of 0.5 was reached. Next, cells were dispensed in sterile round-bottom 96-well plates and were subjected to five different drug treatments: FLC, BPS, DOX, FLC+BPS, and FLC+DOX for the indicated durations. The total volume of each well was 200 µL of SC 2% Gal with a drug concentration of 8 µg/mL for FLC and 25 µM for BPS and DOX. Thirty minutes before the end of the indicated treatment duration, dichlorodihydrofluorescein diacetate (H_2_DCFDA) dye was added with a final concentration of 40 µg/mL, and the plate was incubated in the dark. The guava flow cytometer was used to measure the fluorescence of singlets, filtered using forward scatter height and area, with an excitation and emission wavelength of 485 and 535 nm, respectively.

### qPCR assay

Cells were grown according to the ROS assay and harvested on ice after 7 h of treatment before transferring to extraction buffer (1 mM EDTA, 0.1 M LiCl, 0.1 M Tris-Cl at pH 7.5) with PCI (citrate-buffered, water-equilibrated phenol at pH 4.2, chloroform and isoamyl alcohol at 25:24:1) containing 1% SDS. Cells were disrupted using glass beads and a fest prep desiccator and cooled. Samples were centrifuged at max speed for 10 min before transferring the aqueous upper layer containing the RNA to a new Eppendorf. Samples are stored at −20°C overnight with 1/10 volume of 40% Kac and 2 volumes of 100% EtOH, before a final centrifugation, which pellets the RNA fraction. The samples were treated with DNase before producing cDNA using the iScript cDNAse kit. qPCRs were performed using the GoTaq qPCR method on a StepOnePlus machine before analysis in qBasePlus.

### Statistics

One-way ANOVA with Tukey’s multiple comparison correction and two-way ANOVA with Dunnett’s multiple testing correction were performed whenever either multiple strains were compared or multiple strains with multiple conditions. When single strains are compared against a set theoretical value (Fig. 5A), a one-sample *t*-test is performed. At least three biological transformants are present for each experiment. Statistical significance is indicated using asterisks as follows: *P* < 0.05 (*), *P* < 0.01 (**), *P* < 0.001 (***), *P* < 0.0001 (****).

## Data Availability

All the data described in this manuscript are available from the authors.

## SUPPLEMENTAL MATERIAL

Data set S1 mbio.00416-26-s0001.xlsx Extracted proteomics data set.

Fig. S1 mbio.00416-26-s0002.tiff Synergy assessment of fluconazole and DOX to intracellular iron homeostasis mutants.

Fig. S2 mbio.00416-26-s0003.tiff Synergy assessment of fluconazole and DOX to intracellular iron homeostasis mutants.

Fig. S3 mbio.00416-26-s0004.tif ROS accumulation and ACT1 promoter activity during DOX and FLC therapy.

Fig. S4 mbio.00416-26-s0005.tiff Sod2 overexpression alters the DOX + FLC synergy.

Fig. S5 mbio.00416-26-s0006.tiff Intracellular iron chelation by Bphen does not result in a cidal combination therapy with FLC.

Fig. S6 mbio.00416-26-s0007.tiff Synergy assessment of fluconazole and doxycycline to the wild type and SMF3 deletion strain under anaerobic conditions.

Supplemental tables mbio.00416-26-s0008.xlsx Tables S1 to S5.

ASM does not own the copyrights to Supplemental Material that may be linked to, or accessed through, an article. The authors have granted ASM a non-exclusive, world-wide license to publish the Supplemental Material files. Please contact the corresponding author directly for reuse.
