# Supplementary figures and images for: Vacuolar iron export alters the synergy between doxycycline and fluconazole by affecting cidal ROS levels in Candida albicans
Source: mBio. 2026 Apr 20;17(5):e00416-26. doi: 10.1128/mbio.00416-26 (PMC13170289; doi:10.1128/mbio.00416-26)

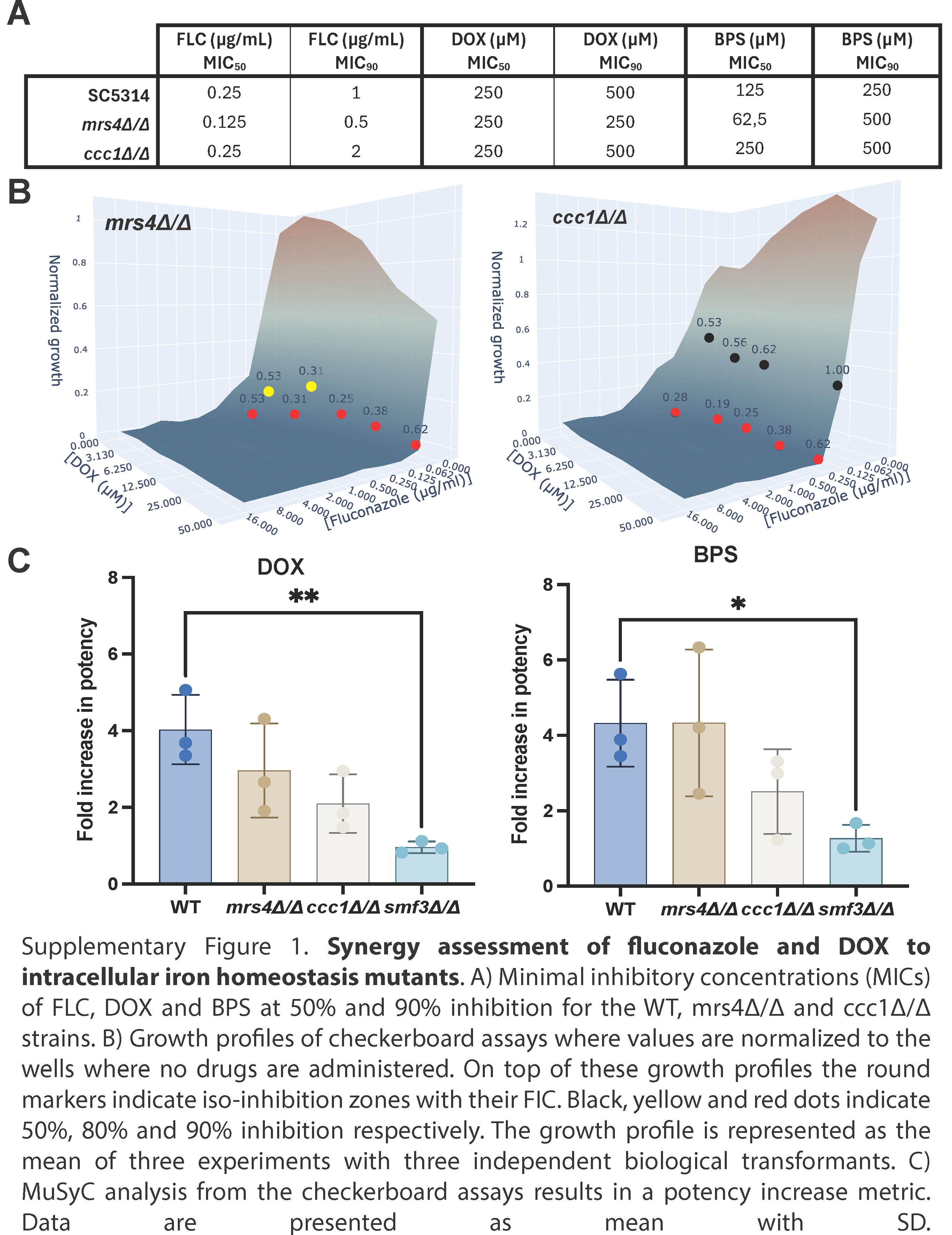

Supplement: Fig. S1 — Synergy assessment of fluconazole and DOX to intracellular iron homeostasis mutants. [file mbio.00416-26-s0002.tiff]

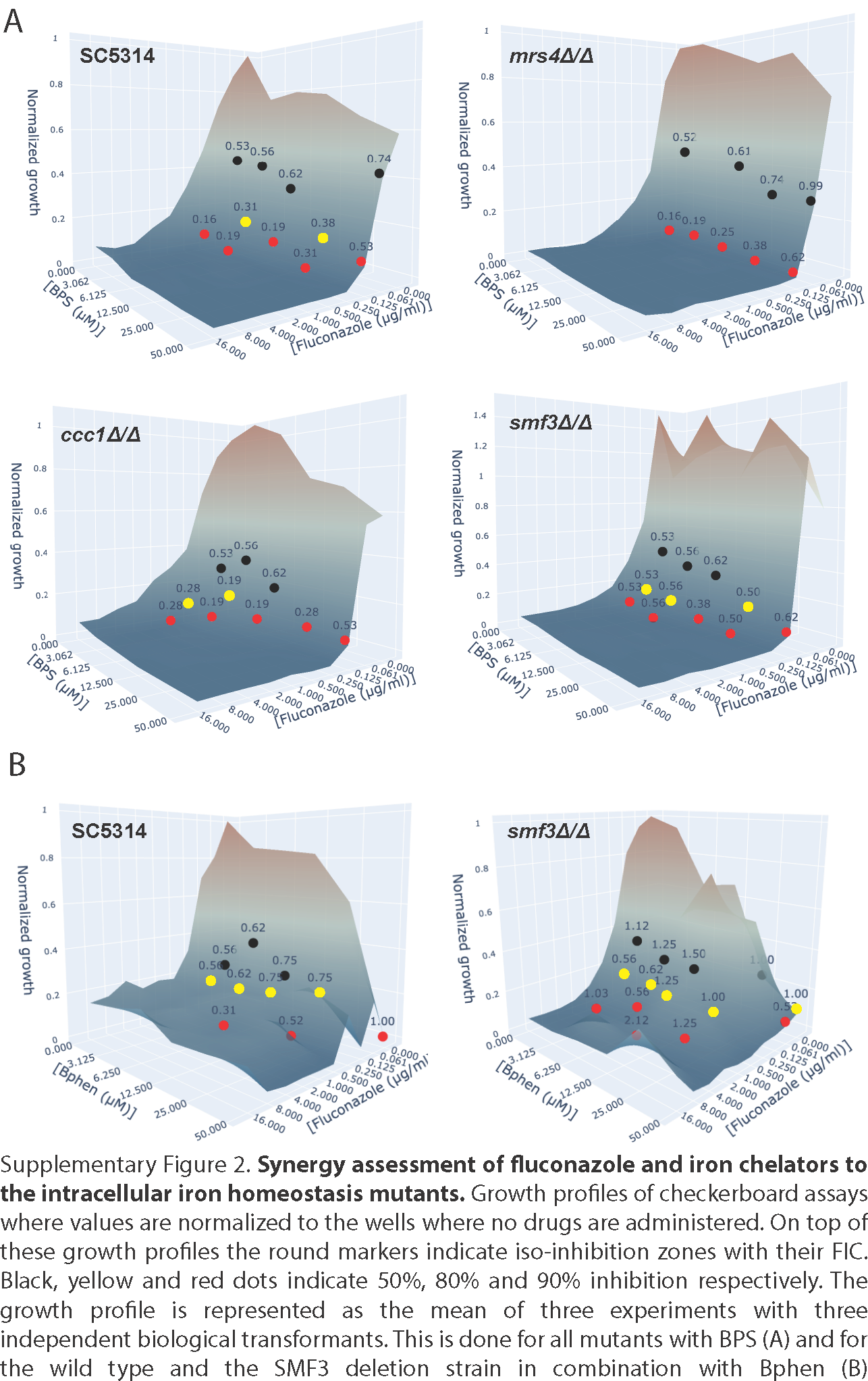

Supplement: Fig. S2 — Synergy assessment of fluconazole and DOX to intracellular iron homeostasis mutants. [file mbio.00416-26-s0003.tiff]

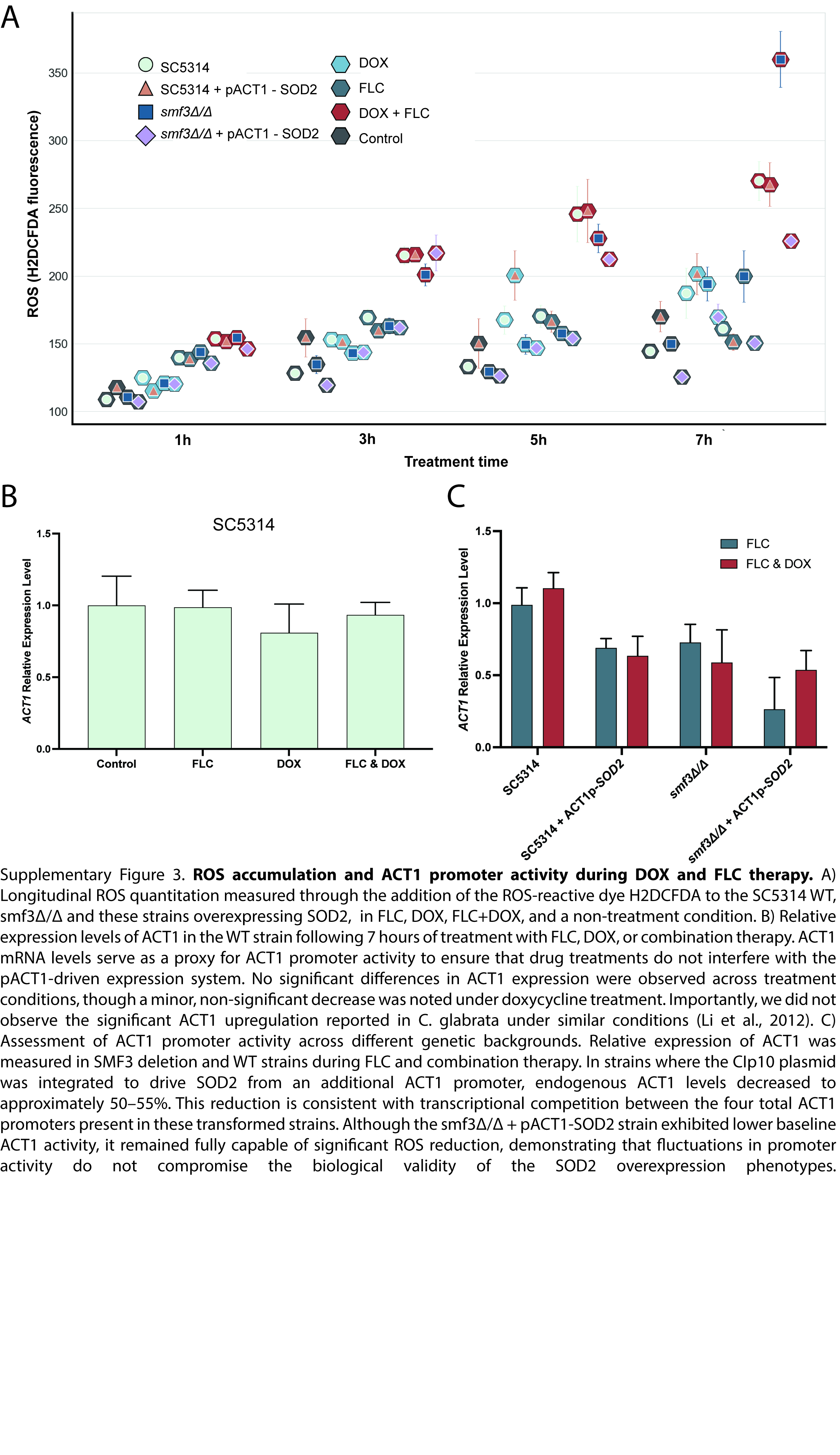

Supplement: Fig. S3 — ROS accumulation and ACT1 promoter activity during DOX and FLC therapy. [file mbio.00416-26-s0004.tif]

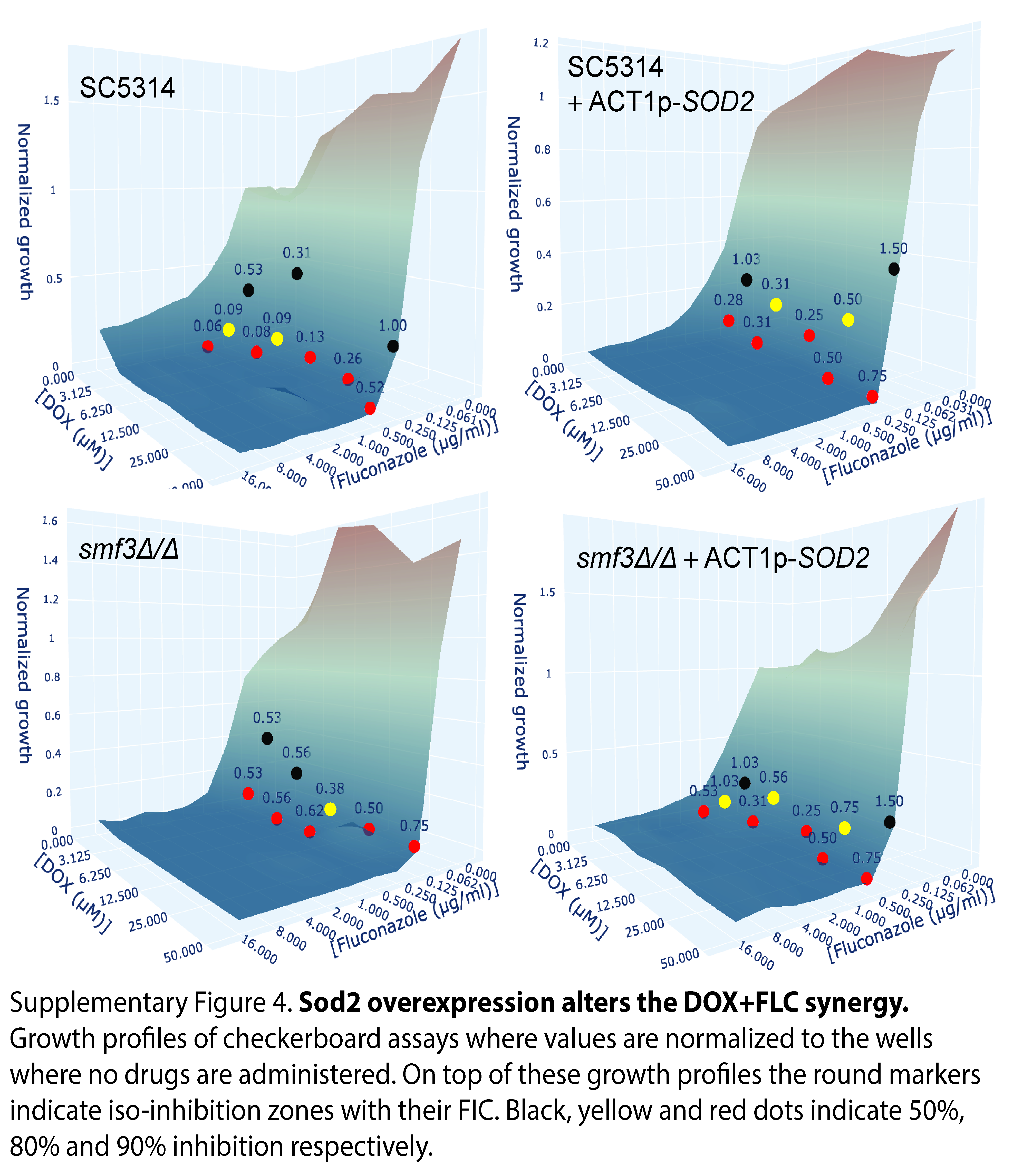

Supplement: Fig. S4 — Sod2 overexpression alters the DOX + FLC synergy. [file mbio.00416-26-s0005.tiff]

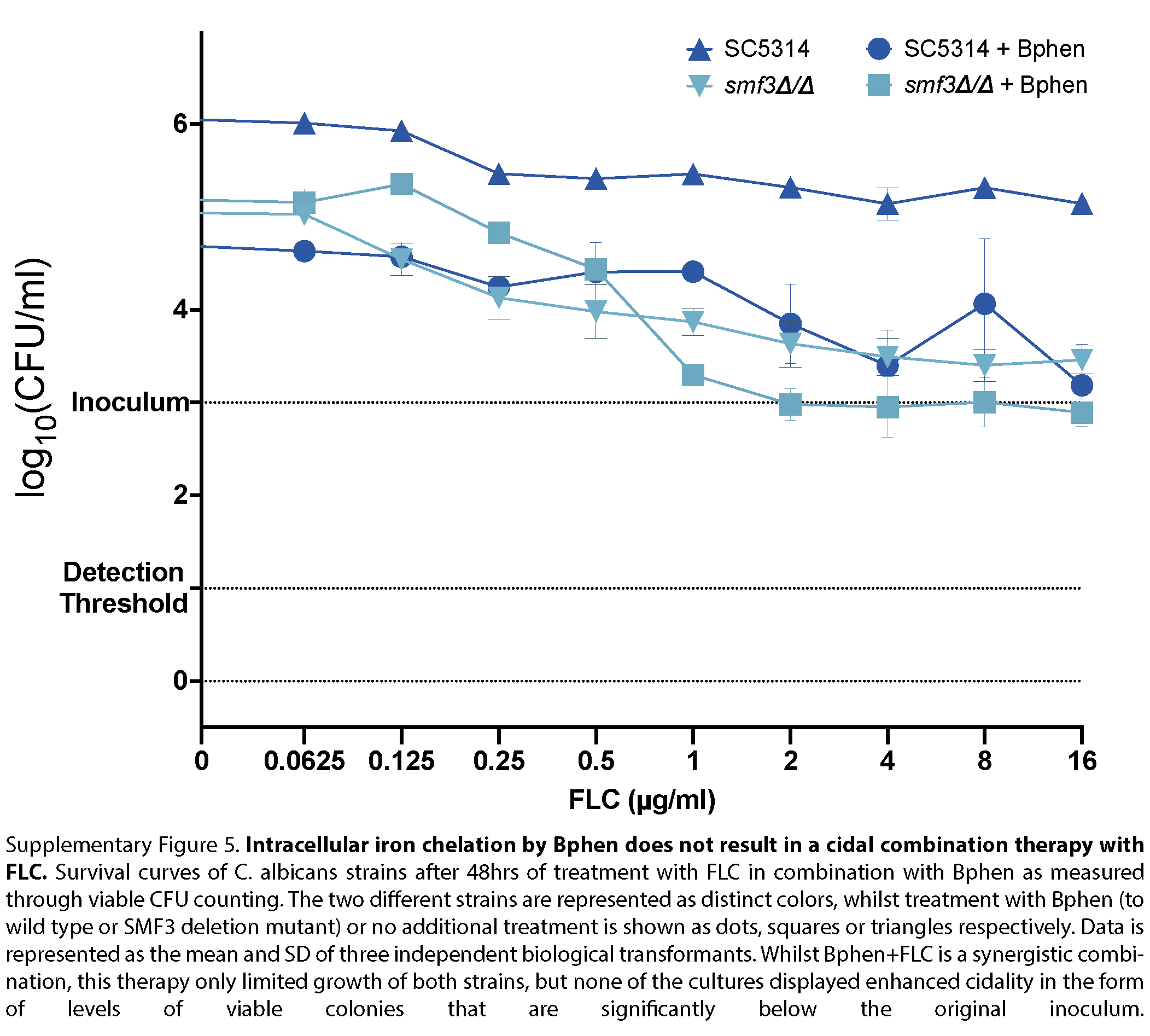

Supplement: Fig. S5 — Intracellular iron chelation by Bphen does not result in a cidal combination therapy with FLC. [file mbio.00416-26-s0006.tiff]

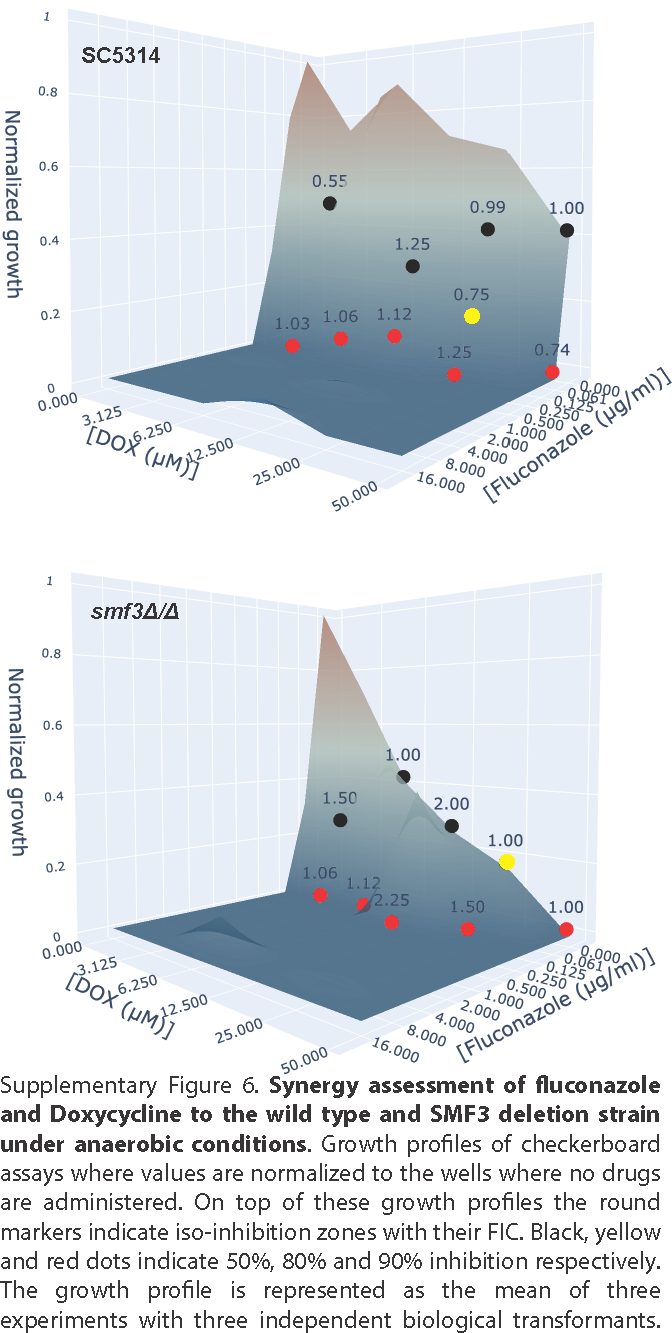

Supplement: Fig. S6 — Synergy assessment of fluconazole and doxycycline to the wild type and SMF3 deletion strain under anaerobic conditions. [file mbio.00416-26-s0007.tiff]
